# Evaluate the value of prolonging the duration of tiopronin for injection administration in preventing hepatotoxicity

**DOI:** 10.1038/s41598-024-54314-3

**Published:** 2024-02-14

**Authors:** Hongye Yang, Mingzhu Lin, Mengxing Liu, Huawei Gu, Dan Li, Yu Shi, Xidong Hou

**Affiliations:** Department of Pharmacy, The Affiliated Anyang Tumor Hospital of Henan University of Science and Technology, Huanbin North Road, Anyang City, 455000 Henan Province China

**Keywords:** Tiopronin for injection (TI), Hepatotoxicity, Gynecological cancer, Prophylaxis, Medical research, Oncology

## Abstract

As part of supportive therapy, prophylaxis with tiopronin for injection (TI) against common hepatotoxicity complications has often been used. However, methods to prevent hepatotoxicity have not been established. Therefore, our study was aimed to find out the relationship between the periods of TI prophylaxis and post-treatment hepatotoxicity, and evaluated the value of prolonging the duration of TI administration in preventing hepatotoxicity. Hepatotoxicity was detected through liver transaminases, bilirubin, alkaline phosphatase, and clinical features of liver insufficiency. Multivariable logistic regressions were conducted to examine the association of the periods of TI prophylaxis and post-treatment hepatotoxicity. Between January 2022 and March 2023, a total of 452 patients with gynecological cancer were enrolled in the study, of which 93 (20.58%) participants were post-treatment hepatotoxicity positive. TI with different prevention days were no significant difference among participants with or without post-treatment hepatotoxicity in crude model (*P* > 0.05). The *P*-value, the odds ratios (OR) and 95% confidence intervals (CI) of participants with TI prophylaxis for 1 day for post-treatment hepatotoxicity were 0.040, 3.534 (1.061–11.765) in fully adjusted model. Past history of hepatotoxicity is a confounding variable, and there was no significant difference for post-treatment hepatotoxicity when stratified by past history of hepatotoxicity (*P* > 0.05). The study indicate that the periods of TI prophylaxis is not associated with post-treatment hepatotoxicity, suggesting that prolonged the periods of TI prophylaxis might be an invalid method for the prevention of post-treatment hepatotoxicity.

## Introduction

Gynecological cancer patients are a group of cancers involving the female reproductive system, that includes cancers of the ovary, cervix, uterine corpus, fallopian tube, vagina and vulva^1^. In 2018, the combined mortality for cervical, uterine and ovarian cancers reached 14%. In 2022, Cancers of the cervix and ovary account, respectively, for the 7th and 9th tumor mortality rate among women in China^2,3^. Current gynecological cancer treatment methods mainly include surgery, chemotherapy, radiotherapy, and the combined chemo-radiation^4–6^. However, prolonged chemotherapy often leads to varying degrees of liver toxicity and relatively specific pathological changes, resulting in decreased liver tolerance and discontinuation of chemotherapy^7–9^. Although there is no consensus on their effectiveness against drug-induced hepatotoxicity, hepatoprotective drugs have often been used empirically in patients with drug-induced hepatotoxicity^10^.

Tiopronin, is a low-molecular-weight thiol derivative of glycine that has been used to treat a variety of conditions. Its –SH moiety can reduce disulfide linkages between oxidized biothiols, like glutathione disulfide, restoring them to their native state. In addition, tiopronin is regarded as an effective chelator of heavy metals such as mercury and copper. Because of these significant antioxidant properties, tiopronin has been used to protect against chemotherapy-induced nephrotoxicity and hepatotoxicity^11^. Tiopronin has also proved effective in perioperative liver function protection. Its preventive role in chemotherapy induced hepatotoxicity is important for cancers^12^.

Some clinicians believe that the duration of TI prophylaxis is associated with post-treatment hepatotoxicity, and the duration of TI prophylaxis should be prolonged when the high hepatotoxicity drugs were used, and/or the duration of chemotherapy drugs is longer, and/or the Past history of hepatotoxicity positive. Thus, we conducted a study in patients with gynecological cancer to explore the association between the periods of TI prophylaxis and post-treatment hepatotoxicity.

## Results

### General characteristics of subjects

A total of 452 individuals were included in this study; 452 (100%) were females, with an average age of 57.00 ± 17.00 years. The clinical and demographic characteristics are shown in Table 1. The proportion of recurrent hepatotoxicity in patients with past history of hepatotoxicity was higher in the post-treatment hepatotoxicity (+) group than in the post-treatment hepatotoxicity (−) group (*P* < 0.05). In addition, combinations of chemotherapeutic drugs and duration of chemotherapy drugs were significant difference in participants with or without post-treatment hepatotoxicity (*P* < 0.05). In contrast, TI with different prevention days were no significant difference among participants with or without post-treatment hepatotoxicity (*P* > 0.05).

**Table 1:** Demographic and clinical characteristics of the participants.

Variables	Total	Post-treatment hepatotoxicity (−)	Post-treatment hepatotoxicity (+)	*P*-value
(n, %)	452	359 (79.42)	93 (20.58)	
Age (years)	57.00 ± 17.00	58.00 ± 17.00	55.00 ± 16.00	0.153
Hypertension (n, %)	149 (32.96)	121 (33.70)	28 (30.11)	0.511
Diabetes mellitus (n, %)	77 (17.04)	63 (17.55)	14 (15.05)	0.568
Hyperlipidemia (n, %)	22 (4.87)	16 (4.46)	6 (6.45)	0.599
Liver underlying disease (n, %)	44 (9.73)	33 (9.19)	11 (11.83)	0.445
Combined chemo-radiation (n, %)	93 (20.58)	74 (20.61)	19 (20.43)	0.969
Past history of hepatotoxicity (+) (n, %)	169 (37.39)	119 (33.15)	50 (53.76)	** < 0.001**
Tumor type (n, %)				0.275
Cervix cancer	210 (46.46)	165 (45.96)	45 (48.39)	
Ovary cancer	182 (40.27)	141 (39.28)	41 (44.09)	
Endometrial cancer	34 (7.52)	29 (8.08)	5 (5.38)	
Other types of gynecological cancer	26 (5.75)	24 (6.69)	2 (2.15)	
Combinations of chemotherapeutic drugs (n, %)				**0.016**
PLPB group	211 (46.68)	175 (48.75)	36 (38.71)	
CPPB group	108 (23.89)	74 (20.61)	34 (36.56)	
PPB group	38 (8.41)	31 (8.64)	7 (7.53)	
OCCD group	95 (21.02)	79 (22.01)	16 (17.20)	
Duration of chemotherapy drugs (n, %)				**0.011**
1 day	147 (32.52)	124 (34.54)	23 (24.73)	
2 days	171 (37.83)	136 (37.88)	35 (37.63)	
3 days	73 (16.15)	48 (13.37)	25 (26.88)	
≥ 4 days	61 (13.50)	51 (14.21)	10 (10.75)	
TI with different prevention days (n, %)				0.692
1 day	25 (5.53)	17 (4.74)	8 (8.60)	
2 days	101 (22.35)	82 (22.84)	19 (20.43)	
3 days	151 (33.41)	121 (33.70)	30 (32.26)	
4 days	98 (21.68)	78 (21.73)	20 (21.51)	
≥ 5 days	77 (17.04)	61 (16.99)	16 (17.20)	

The data were present as medians ± quartiles or n (%). PLPB group, Paclitaxel liposome + platinum ± bevacizumab; CPPB group, Combination of paclitaxel for injection (Albumin Bound) + platinum ± bevacizumab; PPB group, Paclitaxel + platinum ± bevacizumab; OCCD group, other combinations of chemotherapy drugs; (+), positive; (−), negative. Underlying liver diseases include fatty liver, hepatic cyst, hepatic hemangioma, and hepatic nodule. Bold type-statistically significant.

### Association between the periods of TI prophylaxis and post-treatment hepatotoxicity

Multivariable logistic regressions were conducted to examine the association of the periods of TI prophylaxis and post-treatment hepatotoxicity. As shown in Table 2, the *P*-value, the OR and 95% CI of participants with TI prophylaxis for 1 day for post-treatment hepatotoxicity were 0.254, 1.794 (0.657–4.899) in the crude Model. After further adjusting for duration of chemotherapy drugs, combinations of chemotherapeutic drugs, and past history of hepatotoxicity, the* P*-value, the OR and 95% CI of participants with TI prophylaxis for 1 day for post-treatment hepatotoxicity were 0.040, 3.534 (1.061–11.765) (Model 3).

**Table 2:** Association between periods of TI prophylaxis and post-treatment hepatotoxicity.

	OR (95% CI)	*P*-value
Crude model		
TI prophylaxis for ≥ 5 days		0.703
TI prophylaxis for 1 day	1.794 (0.657–4.899)	0.254
TI prophylaxis for 2 days	0.883 (0.420–1.857)	0.744
TI prophylaxis for 3 days	0.945 (0.479–1.866)	0.871
TI prophylaxis for 4 days	0.978 (0.467–2.044)	0.952
Model 1		
TI prophylaxis for ≥ 5 days		0.428
TI prophylaxis for 1 day	3.186 (0.978–10.386)	0.055
TI prophylaxis for 2 days	1.740 (0.618–4.898)	0.294
TI prophylaxis for 3 days	1.600 (0.662–3.865)	0.296
TI prophylaxis for 4 days	1.338 (0.593–3.017)	0.483
Model 2		
TI prophylaxis for ≥ 5 days		0.432
TI prophylaxis for 1 day	3.175 (0.972–10.367)	0.056
TI prophylaxis for 2 days	1.732 (0.613–4.890)	0.300
TI prophylaxis for 3 days	1.596 (0.660–3.861)	0.299
TI prophylaxis for 4 days	1.344 (0.591–3.013)	0.488
Model 3		
TI prophylaxis for ≥ 5 days		0.292
TI prophylaxis for 1 day	3.534 (1.061–11.765)	**0.040**
TI prophylaxis for 2 days	2.067 (0.722–5.921)	0.176
TI prophylaxis for 3 days	1.580 (0.648–3.853)	0.314
TI prophylaxis for 4 days	1.246 (0.547–2.842)	0.601

Model 1: adjusted for duration of chemotherapy drugs; Model 2: adjusted for duration of chemotherapy drugs and combinations of chemotherapeutic drugs; Model 3: adjusted for duration of chemotherapy drugs, combinations of chemotherapeutic drugs, and past history of hepatotoxicity. Bold type-statistically significant.

### Analysis of confounding variables

Multivariate logistic regression was used to analyze the confounding variables for post-treatment hepatotoxicity. As is shown in Table 3, past history of hepatotoxicity is a confounding variable.

**Table 3:** Analysis of confounding variables.

Variables	OR (95% CI)	*P*-value
Duration of chemotherapy drugs	1.143 (0.907–1.439)	0.257
Combinations of chemotherapeutic drugs	1.007 (0.824–1.232)	0.943
Past history of hepatotoxicity	2.307 (1.447–3.676)	** < 0.001**

Bold type-statistically significant.

### Sensitivity analysis in previous hepatotoxicity of patients groups

As shown in Table 4, stratified analyses were performed to explore the association between the periods of TI prophylaxis and post-treatment hepatotoxicity. Stratified analyses were made for past history of hepatotoxicity. Participants were divided into two groups according to past history of hepatotoxicity: past history of hepatotoxicity (+) group and past history of hepatotoxicity (−) group. After adjusting for duration of chemotherapy drugs and combinations of chemotherapeutic drugs, there was no significant difference for post-treatment hepatotoxicity when stratified by past history of hepatotoxicity (*P* > 0.05).

**Table 4:** Relationship between the periods of TI prophylaxis and post-treatment hepatotoxicity by past history of hepatotoxicity.

	Crude model	Model 1	Model 2
Past history of hepatotoxicity (−) *P*-value/OR (95% CI)			
TI prophylaxis for ≥ 5 days	0.451	0.436	0.406
TI prophylaxis for 1 day	0.282/2.031 (0.558–7.388)	0.098/3.744 (0.783–17.892)	0.087/3.931 (0.821–18.811)
TI prophylaxis for 2 days	0.518/0.717 (0.261–1.968)	0.593/1.482 (0.350–6.267)	0.562/1.530 (0.363–6.442)
TI prophylaxis for 3 days	0.458/0.688 (0.256–1.847)	0.766/1.214 (0.339–4.350)	0.749/1.231 (0,344–4.404)
TI prophylaxis for 4 days	0.970/1.020 (0.358–2.905)	0.528/1.455 (0.453–4.673)	0.513/1.476 (0.460–4.732)
Past history of hepatotoxicity (+) *P*-value/OR (95% CI)			
TI prophylaxis for ≥ 5 days	0.720	0.487	0.516
TI prophylaxis for 1 day	0.551/1.650 (0.319–8.542)	0.281/2.809 (0.429–18.392)	0.287/2.790 (0.422–18.428)
TI prophylaxis for 2 days	0.338/1.768 (0.552–5.665)	0.136/3.250 (0.689–15.324)	0.163/3.058 (0.636–14.705)
TI prophylaxis for 3 days	0.695/1.215 (0.459–3.214)	0.298/1.949 (0.555–6.846)	0.321/1.898 (0.535–6.732)
TI prophylaxis for 4 days	0.786/0.864 (0.301–2.483)	0.845/1.122 (0.356–3.531)	0.886/1.088 (0.343–3.456)

Model 1: adjusted for duration of chemotherapy drugs; Model 2: adjusted for duration of chemotherapy drugs and combinations of chemotherapeutic drugs. (+), positive ; (−), negative.

## Discussion

In this study, the definitions of hepatotoxicity are as follows, hepatotoxicity is injury to the liver associated with impaired liver function (elevated bilirubin) cause by a foreign substance^13^. Based on the elevation of liver transaminases, bilirubin, alkaline phosphatase, and clinical features of liver insufficiency, the World Health Organization has developed liver toxicity criteria that classifies hepatic damage as one of five grades (0–4)^14^.

TI approved for fatty liver and drug-induced liver injury in China. One publication reported that tiopronin may act as a glutathione peroxidase (GPX) inhibitor to selectively kill multi-drug resistant (MDR) cancer cells. Another reported that MDR overexpressing cancer cells were more sensitive to tiopronin than native cells. Thus, tiopronin could find an additional indication in cancer therapy^15^. Tiopronin is often used as an empirical prophylactic drug in the treatment of gynecological cancer patients. Nevertheless, for all we know, few studies have reported the usage and dosage of TI prophylaxis to prevent hepatotoxicity. Some clinicians empirically believe that prolonged the periods of TI prophylaxis is more effective in preventing hepatotoxicity. From this perspective, it has valuable to study the relationship between the periods of TI prophylaxis and post-treatment hepatotoxicity.

Many risk factors are associated with hepatotoxicity. In particular, several studies have reported a positive association between viral hepatitis and hepatotoxicity^16,17^, therefore we excluded those with viral hepatitis to eliminate the possibility of hepatotoxicity occurring other than elevated liver enzymes. Chemotherapy and radiotherapy are the two main approaches for cancer treatment besides surgery. The combination of chemotherapy and radiotherapy towards more efficient drug delivery^18^. But both treatments have the potential to cause liver toxicity in patients. Some chemotherapeutic drugs have been proven to be Hepatotoxic^19–25^. Low hepatotoxicity has been reported in children after irradiation of various abdominal and thoracic tumors^26^. The combination of radiotherapy and chemotherapy may increase the risk of liver toxicity. In this research, patients were included a combination of chemotherapy and radiotherapy, and the patient experienced low hepatotoxicity after treatment: elevated liver transaminase < 3ULN (Upper limit of normal), and elevated bilirubin < 2.5ULN. Thus, instead of using the criteria for drug hepatotoxicity, we used the criteria for hepatotoxicity established by the World Health Organization. Hepatotoxicity was not reported among the adverse reactions of bevacizumab^27,28^, therefore, bevacizumab is not necessary for grouping chemotherapy drugs. CTLA-4 and PD-1 immune checkpoint inhibitors can cause immune-related hepatotoxicity^24,25^, however, the mechanism of immune-related hepatotoxicity is different from that of chemotherapeutic drugs, so immune checkpoint inhibitors are included in other chemotherapeutic drug groups.

The present study has some limitations. First, this study was a retrospective study, and most of the hepatotoxicity in patients after treatment was grade 1. Therefore, hepatotoxicity was not graded. Second, due to the limited number of applicable patients, we only selected patients with gynecological cancer, patients with other cancers such as breast cancer were not included in this study. Third, TI has been used prophylactically in clinical practice for patients treated to interrupt treatment due to concerns about hepatotoxicity. This fact makes it a lack of data studies on the relationship between hepatotoxicity prevention with and without TI. Despite the above limitations, we believe this study has valuable implications for clinical practice.

In conclusion. The study indicate that the periods of TI prophylaxis is not associated with post-treatment hepatotoxicity, suggesting that prolonged the periods of TI prophylaxis might be an invalid method for the prevention of post-treatment hepatotoxicity.

## Methods

### Study the population and the treatment

This is a retrospective study. Between January 2022 and March 2023, a total of 3144 patients were assessed for eligibility at the Anyang Tumor Hospital. Of these, 452 patients with gynecological cancer were enrolled in the study and subsequently analyzed. The criteria for enrollment in this study were as follows: (1) The patients had no history of alcoholism, smoking or hepatitis. (2) The liver function test of the patient was normal before treatment, and there were no clinical symptoms of liver insufficiency. (3) Patients were treated with chemotherapy or combined chemo-radiation. TI was given prophylactically (0.2 g/day) when patients were treated with chemotherapy or concurrent chemoradiotherapy.

### Data sources

The patients’ data was collected and sorted from our hospital’s electronic database, which contains prior medical records and postoperative follow-up information. Data obtained from medical records included tumor type, treatment method, patient's underlying disease, combinations of chemotherapeutic drugs, duration of chemotherapy drugs, Patient prognosis, duration of TI prophylactic medication, biochemical parameter values and clinical characteristics of patients' alanine aminotransferase (ALT), aspartate aminotransferase (AST), alkaline phosphatase (ALP) and total bilirubin (TBIL).

### Statistical analysis

Categorical variables were described as percentage (%) and compared by Chi-square tests. The Shapiro–Wilk W test was used for continuous variables. Non-normal distributed continuous variables were described as the medians ± quartiles and compared with the Mann–Whitney U test. the multivariable logistic regression models were used to calculate the odds ratio (OR) and 95% confidence interval (95% CI) for post-treatment hepatotoxicity. Adjustments were made for 3 variables (duration of chemotherapy drugs, combinations of chemotherapeutic drugs, and past history of hepatotoxicity), and past history of hepatotoxicity was identified as potential confounders of the risk factors for post-treatment hepatotoxicity. Sensitivity analyses were used to estimate the association between the periods of TI prophylaxis and post-treatment hepatotoxicity, stratified by past history of hepatotoxicity. Statistical analyses were performed using SPSS software (version 20.0, SPSS Inc, Chicago, IL, USA). All statistical tests were 2-sided, and significance levels were 0.05.

### Ethical approval

The protocol for this research project has been approved by a suitably constituted Ethics Committee of the institution, and it conforms to the provisions of the Declaration of Helsinki. Committee of Anyang Tumor Hospital, Approval No. 2023WZ21K01.

### Informed consent

This study is a retrospective study, using medical records obtained from previous clinical treatment, and the data does not include information that can be traced to the subjects' privacy, such as medical record number, ID number, address, and contact information. The exemption from informed consent will not adversely affect the rights and health of the subject. Therefore, informed consent was exempted by the Ethics Committee of Anyang Tumor Hospital.

## Data Availability

The datasets supporting the conclusions of the current study are available in the Affiliated Anyang Tumor Hospital of Henan University of Science and Technology, and the datasets will be shared with the corresponding author upon reasonable request.

## Abbreviations

TI: Tiopronin for injection
GPX: Glutathione peroxidase
MDR: Multi-drug resistant
ALT: Alanine aminotransferase
AST: Aspartate aminotransferase
ALP: Alkaline phosphatase
TBIL: Total bilirubin
ULN: Upper limit of normal
CTLA-4: Cytotoxic T lymphocyte associated antigen-4
PD-1: Programmed death-1
